# The Structural Dilemma of Bulk Polyethylene: An Intermediary Structure

**DOI:** 10.1371/journal.pone.0006228

**Published:** 2009-07-14

**Authors:** Morteza Laridjani, Pierre Leboucher

**Affiliations:** 1 Laboratoire de Physique des Solides, Université de Paris-Sud, Orsay, France; 2 Laboratoire de Physiologie de la Perception de l'Action, CNRS - Collège de France - UMR 7152, Paris, France; University of Portsmouth, United Kingdom

## Abstract

**Background:**

The Fourier space (reciprocal space) image of bulk polyethylene consists of lines superimposed on the coherent diffuse background. The mixed character of the image indicates the complex nature of these compounds. The inability in detecting full images of reciprocal space of polymeric substances without Compton radiation and the other undesirable diffuse scatterings has misled the structural analysis (structural characterisation) of these materials.

**Principal Findings:**

We propose the use of anomalous diffractometry where, it is possible to obtain a real image of reciprocal space without Compton radiation and other undesirable scatterings. By using classical diffractometry techniques this procedure is not possible. This methodology permitted us to obtain the “Direct Delta function”, in the case of polycrystalline substances that was not previously detected. A new procedure was proposed to interpret the image of reciprocal space of bulk polyethylene. The results show the predominance of the geometry of local order determination compared to the crystal unit cell. The analysis of x-ray diffraction images illustrates that the elementary structural unit is a tetrahedron. This structural unit illustrates the atoms in the network scatter in a coherent diffuse manner. Moreover, the interference function derived from the coherent diffuse scattering dampens out quickly and the degree of randomness is superior to a liquid state. The radial distribution function derived from this interference function shows bond shortening in the tetrahedron configuration. It is this particular effect, which stabilises polyethylene.

**Conclusion:**

Here we show by anomalous diffractometry that the traditional concept of the two-phase or the crystal-defect model is an oversimplification of the complex reality. The exploitation of anomalous diffractometry has illustrated that polyethylene has an intermediate ordered structure.

## Introduction

The definite evidence of polymer atomic arrangement periodicity was discovered in the early 20^th^ century by assessment of x-ray diffraction patterns by Nishikama, N. Mull and P. Scherrer [1], [2], [3]. It was suggested that polymers formed chained molecules that were distributed on lattice points with a rigorous periodicity. This regular atomic distribution with a perfect periodicity is known as a long-range order crystalline structure. According to the extinction rule, for this case, the Fourier space image of the substance consisted of only fine lines or sharp spots.

For the first time C.W. Bunn and T.C. Alcock (1945) [4] reported that the Fourier space image of polyethylene were selective reflections superimposed on the diffuse bands. These authors clearly claim the coexistence of two different states of order and disorder in the same sample. Other investigators followed the idea of coexisting ordered and disordered states in polymers [5]. These authors described the atomic structure of the polymeric substances, according to the concept of two-phase model system.

Each Fourier space image from polyethylene was recognised as a linear parallel chain and suggested to co-exist as two regions in the sample:

i - the first region that is the amorphous region was considered as a heap of disordered atoms without offering an explanation on how these atoms were distributed in the heart of the polymer (polyethylene),
ii - the second region was the portion that reflected the lines, that was supposed as the crystalline region. Here the atomic arrangement essentially required periodicity. Due to this assumption the reflected lines were analysed by structural crystallography method (‘Powder method’). Since the Hanawalt method in 1938 [6] , indexing the ‘Powder Pattern’ resulted in a set of line position (θ)/‘d’ spacings, with a set of their integrated relative intensities [5], [6]. However, the diffuse scattering in the x-ray pattern of polymers consisted of the mixture of coherent and non-coherent radiation such as the Compton radiation and other undesirable scatterings. This resulted in an intense diffuse scattering where the lines were superimposed on the background. Consequently, this strong background would not allow the weak reflected lines near the direct beam, a pre-requisite for structural identification, to appear. Therefore, it is an essential requirement to resolve lines from the diffuse component.

Ruland's method [5], [7], [8] recommended a resolving procedure for the identification of the crystalline fraction (x_c_), but this traditional procedure of resolving x-ray diagrams in two parts was always arbitrary [9], [10], [11], [12].

Moreover, the discovery of the lamellae single crystal (thin platelet) of linear polyethylene [13], [14] with a molecular weight of 10,000 and an orthorhombic unit cell as a unit of periodicity led to the following question;

“How does such a long chain fit in a unit cell?”

It was proposed that this long chain must fold back several times on its self (re-entry), in the unit cell. Two questions arose from this suggestion.

Whether emerging chains fold over into a regular adjacent position (adjacent model) or do they fold in a disordered manner (random fashion).

Two antagonist schools [14] and Flory [15], tried to theoretically determine, which models were closer to reality. However both assumptions were far from experimental reality.

Despite numerous investigations of atomic arrangements of long chain polymers, the nature of coherent diffuse scattering observed on the complete Fourier space image of polyethylene is still not well understood.

The purpose of the current work is to precisely detect the full image of the Fourier space of linear polymer such as bulk polyethylene by using a new prototype of diffractometry. This procedure allows the fine analysis of the Fourier space image and numerically separates the selective reflections from the continuous coherent diffuse scattering. This continuous coherent diffuse scattering is known as the form factor, which was also analysed from the effect of randomness of long chains.

This study provides realistic structural information from which we conclude that the current two-phase system model is inconsistent with the complete Fourier space image. We report here that bulk polyethylene has an intermediate state of order as we showed previously for other complex compounds with similar x-ray patterns such as: archetypal quasi-crystalline materials, Frank-Kasper Phase and non- ideal solid solution alloys [16], [17], [18].

## Materials and Methods

### Materials

#### The Guinier Camera (photographic method)

Fine-focus high intensity x-ray tube (target –Cu) was used in conjunction with curved single crystal as focussing monochromatic and Guinier camera adjusted for transmission in symmetrical geometry recorded to 2θ = 60° and asymmetrical geometry recorded from 2θ = 0° to 2θ = 100°. In this configuration of the camera, the diffracted line is only one side and they are superimposed on the background.

The use of the Guinier camera permitted us to eliminate parasitic scattering and the “fogged” region. With this technique we can raise, the sensitivity of method, contrast, and resolution. Moreover, the modulation of background and eventually small angle scattering can be observed. The x-ray diffraction pattern of bulk polyethylene with a density d = 0.92 g/cm^3^ was obtained in a vacuum with a sensitive photographic film. The sample can be mounted on the rotating specimen holder.

#### X-ray Diffractometry method -Anomalous Diffractometer

The aim of this work was to study the image of Fourier space, which consists of the powder lines superimposed on the coherent diffuse scattering. Thus, to precisely detect the weak scattering, and all undesirable scatterings that require to be removed. This elimination was achieved by using anomalous diffractometer. Anomalous diffractometry, was designed and implemented by M. Laridjani [16]. By utilising anomalous diffractometry a real image of the Fourier space of plastic materials can be obtained.

**Figure 1:**
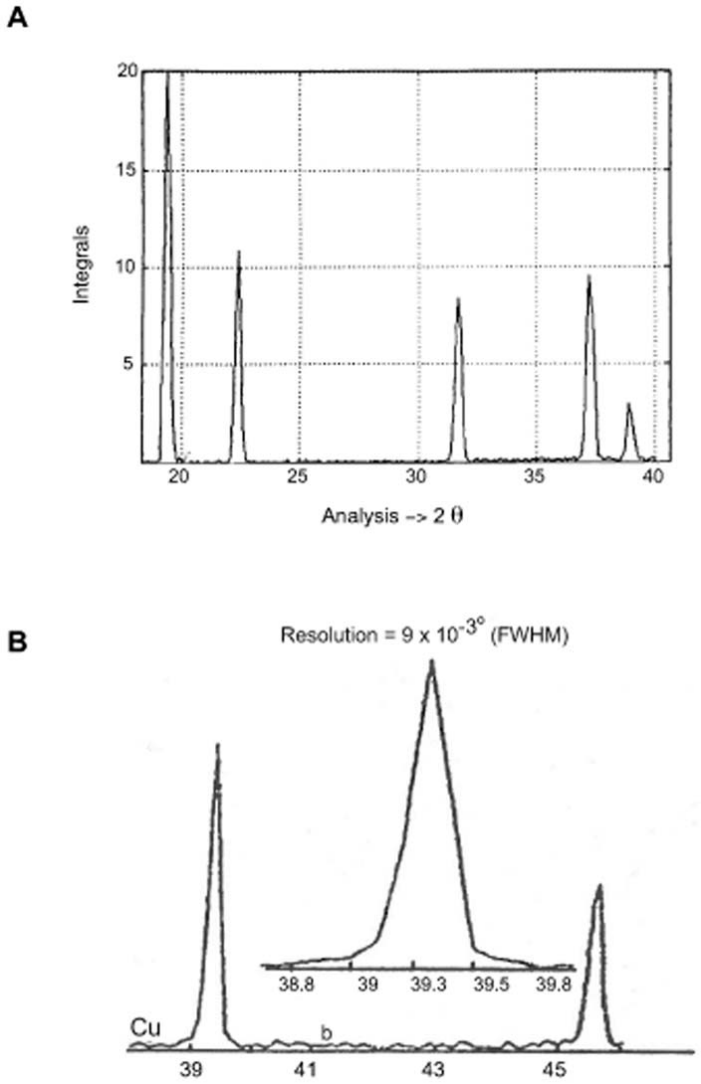
The image of Fourier space of copper polycrystal by anomalous diffractometry. A. This image shows the “extension rule” is true experimentally. B. is the zoomed portion of A (2θ = 30 to 40°). The absence of background is observed between two sharp reflection lines reflected by the lattice planes (hkl) of polycrystalline copper.

This prototype consists of two θ–2θ goniometers with a resolution of 0.001°. The goniometers, installed on a rotating anode generator (Ag or Cu target tube), are adapted to a parallel beam, which is obtained by a special optical system (slit system). This optical system can be finely adjusted to reduce the deviation of the observed diffraction profile from the ideal profile (δ, Diract Delta function), without introducing any background in the case of ideal crystalline materials (Figure 1) [16]. Hence the direct beam, at a very small angle, does not overshadow the scattering from the sample, and this set up also offers the possibility of simultaneously studying very-small angle scattering in the same x-ray diffraction diagram. The data were acquired by new processing software with a prototype lithium-drifted silicon solid-state detector (only 500 g). The detector is connected to a preamplifier and a pulse processor. The pulse processor is a sophisticated signal-processing unit, which provides linear amplification, noise filtering, pulse-pile-up rejection and a lifetime correction. The pulses are collected in a multi-channel analyser interfaced to a computer [19]. The combination of these functions is an essential prerequisite for achieving a precise ‘energy window’ and a high resolution of 150 eV at 8 KeV and 230 eV at 20 KeV [16], [20]. This energy window takes the place of a monochromator in the path of the scattered beam from the sample.

**Figure 2:**
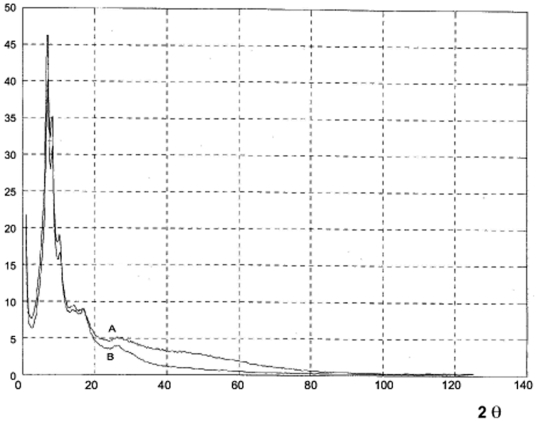
The total image of Fourier space of polyaniline obtained by anomalous diffractometry [25]. A is the x-ray diagram of polyaniline in the presence of diffuse incoherent scattering (Compton radiation) B. This is the real image of the Fourier space of polyaniline obtained by the same scan θ–2θ. In this diagram the Compton radiation was eliminated by the energy window (See Methods and Materials).

Two modes of diffractometry, dispersive and non-dispersive energy, can be used owning to this energy-window system. The non-dispersive mode was the only one used in this study. Here, the monochromatic Kα and Kβ lines of the anode characteristic spectrum, and any wavelength of the white spectrum can be selected in several energy windows and utilized simultaneously. Consequently, in the same experiment, the very low intensity of the coherent continuum diffuse scattering from different K = 4πsinθ/λ can be recorded. This easy wavelength selection makes it feasible to obtain, in the same θ–2θ scan, a small angle scattering diagram (K≥0,001 Å^−1^) and a high-resolution diffraction patterns with different wavelengths and a large K value (K∼28 Å^−1^). The performance of anomalous diffractometry also permits the automatic elimination of Compton and fluorescence radiation (Figure 2), and thereby enhances the quality of the x-ray patterns. In the case of polymers this is unprecedented.

### Sample Preparation

The bulk of low-density polyethylene was cut into the following dimensions; φ = 80×80×2(mm). The same sample was scanned in the reflection and transmission geometry respectively 2θ = 0.2→2θ = 120° and 2θ = 0.2→2θ = 90° by the anomalous diffractometer.

The exact conditions used for producing a number of materials commercially are often only known to the manufactures, especially with regard to the temperature. So precise information cannot always be stated for processing details.

### Methods

### Interpretation of Fourier space image of bulk polyethylene

#### The Resolving procedure of X-ray diagram

**Figure 3:**
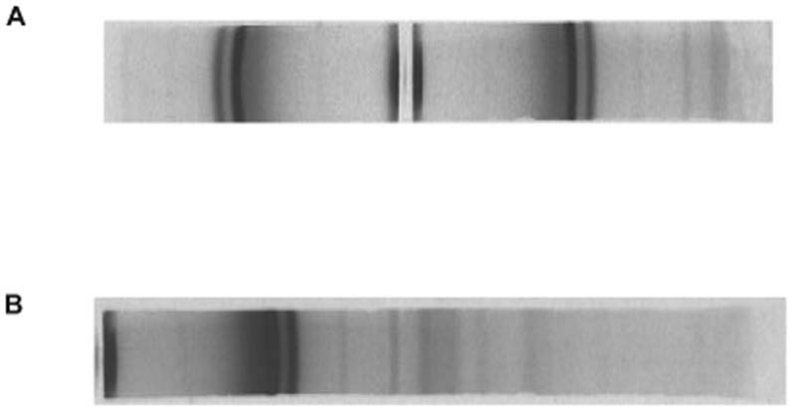
The Guinier pattern of bulk polyethylene. The image of Fourier space of bulk polyethylene was acquired by a focusing camera (Guinier Camera). A- a pattern obtained by the camera with geometrical symmetry. To obtain this image the centre of the camera is placed in the path of the rays (2θ = 60°). B- a pattern obtained by a non-symmetrical camera (2θ = 0 to 2θ = 100). Due to the high dispersion x-ray pattern one gets the impression of observing the x-ray pattern of two different phases at λ = 1.54 Å. The fine diffracted lines at small angle hidden in background are clearly seen in Figure 7F.

The inability of detecting precisely the real image of reciprocal space of polymer samples leads the investigator in polymer science to apply a resolving procedure of x-ray diagrams of polyethylene such as Figure 3A and 3B. The x-ray diagrams have been resolved into two parts: one part was taken, to consider the x-ray image of crystalline polyethylene phase and then indexed. The other part was excluded without knowing its atomic arrangement.

It is this type of resolving procedure misled to the two-phase concept in polymer samples. From the two-phase concept the degree of crystallinity (xc) was defined [5], [10], [21]. The photographic method was used for the first time by Bunn [22], to identify the crystal unit cell of bulk polyethylene.

To understand, the structure identification method of Bunn, we also applied a power method, using a photographic technique (Guinier Camera). Figure 3 shows a high resolution Guinier pattern of bulk polyethylene. This x-ray diagram is the image of Fourier space of sample, indicated clearly a set of broad lines, with width more than 0.2°θ for kα Cu radiation that appeared, side by side, of diffuse rings. At first glance, it appears unlikely to index such a powder line. It is surprising how Bunn identified the structure of the powder lines with a width more then 0.4°θ. It is well known that according to de Wolff's criterion “the pattern with a line width of 0.2°θ or more (for Kα Cu) can be regarded as almost hopeless” to be indexed [23], [24]. Later on a new picture of polymer structure known as the crystal-defect concept was proposed [10].

To identify the crystalline fraction (xc) [5], Ruland's method was recommended. However, the resolving of lines from the background by using the traditional experimental x-ray diagram into two parts was always arbitrary [9], [10], [23], [24]. Ruland's procedure indicated the possibility and difficulties of resolving the selective reflection from the coherent and incoherent diffracted beam. Apart from what he mentioned as a precaution for resolving of two phases, the knowledge of diffraction curve of pure amorphous state with the same chemical composition (chemical structure) was necessary. However, contrary to metallic alloys, obtaining a pure amorphous phase was not a simple task [25]. The latter studies resulted in establishing our new procedures.

#### The separation procedure of selective reflection from the coherent diffuse scattering

**Figure 4:**
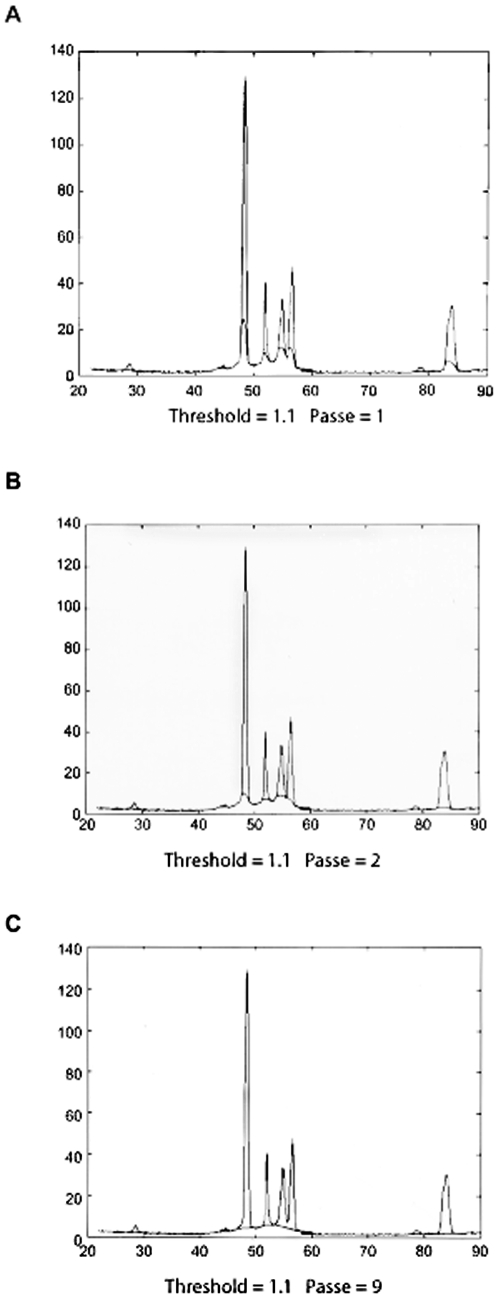
The different stages of separation procedure to obtain lines spectra and coherent diffuse scattering diagram. A. The initial state of separation. B. The intermediate state of separation. C. The final state of separation of lines from the broad halos (Coherent background) [16].

The separation procedure is based on knowing, for a given number of diffracting atoms, the diffracted intensity averaged over the whole reciprocal space. This is independent of the mutual arrangements of atoms. Therefore, an x-ray diagram, which consists of a mixture of line (selective reflections) and diffuse rings (halos) can be analysed by our separation procedure. Using this procedure, the x-ray diagram was numerically separated into rings and selective reflections with respect to the following three hypotheses:

- the law of conservation of coherent intensity scattered by the sample. Meaning that the intensity (I) of each point on the x-ray diagram is the sum of the intensities scattered by diffracting atoms into two parts in form of selective reflections (lines), part (I_1_) and the background, part (I_2_) in the sample. The total intensity:
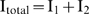

- The intensity of each point of background curve (diffuse rings) should be:
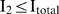

- the intensity of any point of x-ray diagram without lines should be:
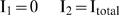

By adopting these three hypotheses, the lineless sections were conserved as a part of the background curve. By linear interpolation, the missing part of background curve and the line diagrams were constructed in order to obtain the coherent diffuse scattering diagram and the line spectrum. MATLAB program [26] was expanded for the computational operations and linear interpolation. Briefly, these operations consisted of selecting a real point on the Ic (2θ) curve and comparing its magnitude with the mean, I, of the previous point. The missing part of diffuse scatter x-ray diagram was constructed point by point up to a very large K. The different steps of the procedure are shown in Figure 4. The resolution and profile of a diffuse scattering diagram obtained by this program was linked to the number of times, the operation was performed and the resolution of the steps for computational averaging.

It is known, that the separation operation is a routinely applied in crystallography to identify some structural details. For example, the Bragg reflection profiles of (hkl) planes, from an x-ray pattern were calculated and the grain size of the crystallite in the substance obtained. Another example was the intensity of diffuse scattering in reciprocal space of random solid solution that was analysed and the effect of clustering defined. The latter was based on the Ewald construction, still valid for position of diffuse zone, outside of direction of selective reflections in reciprocal space) [16], [25], [27]

With the method of separation described above two sets of spectra were obtained: one was a line spectrum and the other ring spectrum or diffuse scattering spectra.

Moreover, both parasite radiation in coherent diffuse scattering -Compton and undesirable background- in θ–2θ scans were eliminated by the diffractomery prototype [17]. We neglected the contribution of thermal diffuse scattering (TDS) compared to coherent diffuse scattering. In fact, the ‘major’ contribution of TDS was due to the inelastic phonon scattering, arising from acoustic modes that were at close vicinity of the Bragg reflections. However, with our experimental resolutions, it was confined inside the Bragg peaks; elsewhere it contributed to the coherent diffuse scattering and was negligible [10], [16].

Analysis of coherent diffuse scattering of Polyethylene-

It is known that a Patterson function [P(r)] is the average of the product of electronic densities at two points (atoms) separated by a vector r in the Patterson space.

*A priori* this function was considered as the “guide for determining crystal structure. [P(r)] was reduced to an atomic distribution function, in the case of disordered atoms and expresses the probability that the centres of two atoms ‘m’ and ‘n’ should lie on vector r [10], [28]. The numerical value of this function was close to unity, when all distances were equally probable. This state was only possible for truly random assemblies of atoms, which meant that a given atom did not have an influence on its neighbour. Such an imaginary state was defined as a perfect gas, which could be considered as the standard state of disorder. The fluctuation of [P(r)] function expresses the local order of a network. If we divided the diffuse scattering curve, I_c_(2θ), of this imaginary state by Nf^2^ then
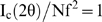



We obtained the interference function equal to 1, which meant that there was an absence of correlation between atoms. In the case of real materials, the intensity, I_N_(K), of diffuse scattering can be approximated by the Debye equation.
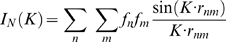
(1)


Here, N is the number of carbon atoms affected by disorder, r_mn_ = (r_mn_−r_n_) and f_n_ is the atomic scattering factor of carbon atoms. If we introduce the atomic density ρ(r), the mean atomic density ρ_0_ and the mean atomic volume v, then ρ_0_ = 1/v.

The Patterson function, [P(r)], is then equal to the ratio of ρ(r)/ρ_0_ and the Debye equation can be rewritten as:
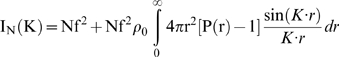
(2)


Defining the reduced interference function as F(K) = K[(I_N_(K)/Nf^2^−1] and using Equation (2), we obtained
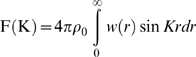



By Fourier-inverting we obtained
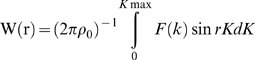
(3)where W(r) = r[P(r)−1]was defined as the reduced radial distribution function.

Equations 1 and 2 were applied to the monatomic components, as in the case of polyethylene [28]. As the x-ray scattering factor of hydrogen was negligible, data could not be obtained from one-dimensional x-ray scattering. Therefore, precise information about interatomic distances involving hydrogen atoms was not obtained.

In practice, the interference function was derived from the experimental data Ic(2θ). Measured intensities were modified according to the absorptions of x-rays by the sample. Consequently, the intensities have to be corrected in order to obtain the intensity function, Ic(2θ). This intensity was only characteristic of the coherent scattering process. This weak absorption correction was performed for angular variations using geometrical arguments. It is known that in the reflection geometry, the absorption corrected factor does not depend upon the diffraction angle; therefore in a relative measurement corrections were unnecessary. Intensity measurements by transmission and reflection geometry were adjusted by overlapping the common angular domains. Moreover, the difficulties in obtaining a high quality experimental intensity, Ic (K) were related directly to the Kmax range. For a large K (Kmax,≥20 Å^−1^) a very high precision was required to detect a very weak diffraction beam by a polymer sample with a weak scattering power (f^2^) . Due to this eliminating an intense Compton radiation by classical diffractometry was a delicate procedure. These major difficulties–the elimination of Compton radiation experimentally- and other classical minor obstacles were solved by anomalous diffractometry. This method permitted to obtain a near perfect reduced interference function, derived from these data using the classical procedure of normalisation. This procedure of normalisation was originally introduced by Kaplow *et. al* .[29] and afterwards developed in the Guinier School to study the structure of disordered materials (see [30], [31]. Normalisation consists of dividing the experimental intensity by the calculated gaseous scattering intensity and adjusted to the large value of K≥20 Å^−1^. Small errors with were detected and corrected by taking into account the low r (Å) contribution to the reduced radial distribution function obtained by Fourier transformation. By this method the experimental intensity was analytically corrected. According to this argument the interatomic distances were not smaller than the nearest neighbouring distance. This indicated that the curve below the first peak of the reduced radial distribution function, W(r), had a slope of −1. That is the probability of finding an atom at distance, r, from the reference atom was zero. P(r) = 0. Therefore, the accuracy of the experimental data could most easily be determined by the behaviour of the curve below the first peak. If the curve in front of the first true peak had an acceptable characteristic, as mentioned above, the data could be considered to be of reasonable quality. We succeeded in obtaining a distinct total radial distribution function and partial atomic distribution function various metallic alloys and polymers [25], [30], [31]. Therefore, the mean density, ρ_0_, of Equation 3 was calculated and compared to the macroscopic density measurements obtained by other methods.

## Results and Discussion

### Analysis and interpretation of Fourier space image (the whole reciprocal space image)

**Figure 5:**
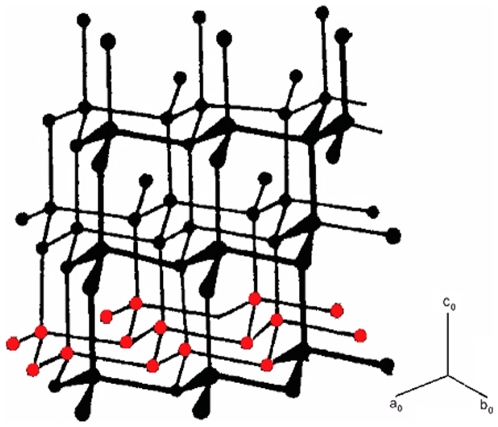
The crystalline chain model of diamond with different disposition of its structure. Here the macromolecule chains of carbon are in parallel. The chain crystalline model of polyethylene was modelled from this structure [21].

Two facts are of note:

a-Background information about structural determination of polyethylene by the powder method
b-The indexing problem of x-ray powder pattern

a. Background information about structural determination of polyethylene by the powder method: According to literature the ‘configuration’ of polyethylene chain [CH_2_]_n_ was curved out from the structure of diamond which obeys the (8-N) rule. Here the carbon atoms were arranged by two interpenetration faced-centered lattices and each atom of lattice B was surrounded by four atoms of lattice A and vice a versa. In the atomic network of diamond, each atom was arranged with a tetrahedral configuration. This could be considered as the geometry of the local topology (Figure 5).

The atomic close packing model defined the atomic radius of carbon, as half of the closest distance of neighbouring atoms in the crystal lattice. The lattice parameter of diamond was reported to be a_o_ = 3.5680 Å, and the interatomic distance of c–c = 1.5433 Å where half of this value was considered as the atomic radius or the Goldschmidt radius. In the atomic close packed model of diamond, the carbon atom with homopolar binding obeyed the (8–N) rule. In this configuration diamond was regarded as a substance where its carbon atoms were formed by close packing to set up the symmetry of its structure. This is known as the atomic closed packed model.

Another view on the whole network of the diamond crystal indicated an immense covalent molecule where, each atom built an octet of electrons by sharing one electron with each of its four neighbours (see Figure 5). These same ‘tetrahedral valencies” were associated to carbon atoms in organic chemistry which resulted in defining configuration or chemical structure. The four covalent bonds of diamond were directed towards the corners of tetrahedron.

This macromolecule chain of carbon atoms was the other disposition of diamond where each macromolecule chain was disposed parallel to the other chain in space. This model is the crystal chain model.

Therefore, the structure of diamond was defined by a network of crystal chains where carbon atoms, inside the unit cell, zigzag along its path in 3D space (Figure 5). In this network, the interatomic distances of carbon-carbon atoms or the bond length (l) was the same as 1.54334 Å and in the chain the bond angle (θ) was 109°28'. It is only the carbon in the diamond network that has these latter characteristics.

This structural duality i.e. the crystal chain and atomic close packing model, was partially “adapted” for the backbone of polyethylene since a single crystal structure of long chains such as normal paraffin was determined in 1928 by Muller [32]. This structural identification of thin flacks of (C_29_H_60_) led Muller to propose a crystal chain model or crystal molecule where the crystal molecule chain was considered as a rod packed laterally close together.

This model was similar to the carbon crystal chain model in the diamond network if we considered the network of paraffin chains to be packed only in the C_0_ direction (Figure 5). However the crystal lattice of the polymer was identified as an orthorhombic with lattice parameters a_0_ = 7.64 Å, b_0_ = 4.480 Å and c_0_ = 2.543 Å. For a single crystal of normal paraffin these lateral rods have definite cross sections that depict the crystal chain with repeat distances of c_0_ = 2.543 Å in the interior of orthorhombic unit cell [32].

In the Muller model, the long chain backbone crystal molecules were formed by carbon atoms zigzagging laterally in the plane as in the case of diamond network.

b- The indexing problem of x-ray powder pattern: The key to understand polymer structure was to distinguish the d (hkl) from d. Light diffracts by optical gratings under the condition nλ = 2dsinθ. Here d is the interval where the lines repeat the positions of the spectra dependant upon d. This condition (nλ = 2dsinθ) was adopted by Bragg for scattering waves from points arranged periodically in space (lattice-space). Therefore the scattering wave from all the points reinforced each other and the most intense beam was diffracted. At other angles of incidence destruction interference will make the beam intensity vanishingly small. This way of considering the condition for diffraction is convenient and the diffracted beam are often termed “reflection” of x-rays from (hkl) family of lattice plans. Therefore under conditions where the atomic network was arranged periodically the reflection condition would be; nλ = 2d_(hkl)_ sinθ, where d in this case is the interplanar spacing of the (hkl) family planes. This equation is the Bragg law. It should be noted that this law is only true when diffracted beams from the atoms in the lattice-space totally vanished between the reflection lines (Bragg lines- see Figure 1). Therefore when the x-ray diagram of a substance consisted of selective lines superimposed on the coherent diffuse scattering, the substance in this case could not be considered as a crystal. Nevertheless, d, was calculated. This only led to the direction where the diffraction beam was reconstructed. Moreover, the nature of the unit of periodic pattern in space (known as the unit cell) was highly important for determining the relative intensities of each reflection line. Therefore the basic principle involved in the structural determination was to precisely know the position (θ) and the relative intensity (I) of the powder lines. Bunn examined [22] by powder method, a series of oriented bulk polyethylenes by rolling each piece of sheet with different number of carbon atoms from n = 20 to n = 3000. He compared the positions (θ) and relative intensities (I) of powder lines of each x-ray pattern obtained of samples with different “n”. Surprisingly, he concluded “the x-ray pattern of different samples was practically constant for molecules over 130 atoms long”. To index the powder patterns he supposed an orthorhombic symmetry with lattice parameters of a_0_ = 7.64 Å, b_0_ = 4.480 Å and c_0_ = 2.543 Å. By this structural assimilation it was concluded that the crystal chain model of Muller for normal paraffin single crystal was consistent with the x-ray diffraction pattern of bulk polyethylene despite the different degrees of polymerisation (n). With such an assumption c_0_ = 2.543 Å was considered as an axis along molecular chain with a repeat distance of 2.543 Å and bond chain angle θ = 112°. Using this assumption it was concluded that, the backbone of bulk polyethylene with bond length l = 1.53 Å can be assumed that the planar zigzag arrangement was the only reasonable arrangement for carbon-atom backbone of bulk polyethylene independent of its molecular weight.

However, it is known that the conformation of the structural model, obtained by the powder method, was based on the agreement of observed and calculated intensities of powder lines, reflected by lattice planes (hkl). The close inspection of Bunn's structural identification was necessary to state the agreement. Table 1, shows Bunn's structural identification, indicating discrepancies between measured and calculated intensities. These were only two powder lines [d [_33_] = 4.106 and d[_17_] = 3.693 Å] between 24 diffracted lines where the calculated and observed intensities matched together. The approach of the crystal structure identification suggested by Bunn was tried without success. One of the possible reasons for the discrepancies may be attributed to the presence of a strong coherent or incoherent background. This is of note as the measured intensities were defined as the peak to background ratio [34], [35].

**Table 1:** Referring to Bunn, (d_hkl_) spacings are calculated from the crystalline model [22] and (I) intensity is measured and calculated by Bunn [4], [22].

This work			Bunn				
2 θ	I	d	d _hkl meas._	d_ hkl cal_	I		hkl
*	*	*			measure	calc	
11.33		7.8					
19.799	3018	4.478					
21.399	40389	4.147	4.106	4.102	4400	4400	110
23.799	7317	3.73	3.696	3.696	1160	1165	200
29.799	426	2.93	2.964	2.956	48	35	210
36.2	1119	2.478	2.467	2.467	226	100	020
38.2	129	2.35	2.346	2.340	18	10	120
39.8	498	2.252	2.252	2.954	105	73	011
40.6	450	2.219	2.202	2.202	248	100	310
41.597	320	2.17	2.162	2.156	75	48	111
43.0	315	2.10	2.088	2.089	140	94	201
43.799	298	2.054	2.067	2.050	70	175	220
46.799	259	1.939	1.925	1.924	60	70	211
49.2	54	1.849	1.849	1.848	(5)	41	400
52.8	376	1.731	1.72	1.72	(5)	56	320, 410
54.6	127	1.678	1.665	1.663	10	134	
57.2	93	1.608	1.598	1.596	(2)	28	130
61.2	69	1.5126					
62	59	1.495	1.499	1.50	(1)	20	230
64.4	52	1.4449	1.434	1.435	(0)	9	510
66	28	1.41					
66.4	31	1.40					
			1.38	1.379	(0)		
			1.294	1.292	231		
			1.267	1.267	002		
			1.234	1.136	511		
			1.210	1.210	112		
			1.123	1.117	022		
			1.098	1.098	530		

In this work, (d) spacings are calculated as a classical calculation of resolving power of ruled gratings. (I) and (2θ) respectively are the intensity and the positions of selective reflections after separation from coherent background.

* Before the first line reported by Bunn there at least 25 reflections (see Figure 7D–F).

Other investigators who followed Bunn by utilising the same classical methodology for identifying the structure of bulk oriented polyethylene obtained the x-ray powder pattern. Some proposed the same crystal structure with extra lines and others suggested variations in the unit cell dimensions

The discrepancies between the calculated and measured intensities set the basis for us to obtain the real image of reciprocal space, of bulk polyethylene by anomalous diffractometery. It was only possible to obtain a real image without Compton radiation by anomalous diffractometery. Hence it was more convenient to compare the experimental with the calculated data for determining the crystalline structure.

### Analysis of Fourier space image (whole reciprocal space) of non-oriented bulk polyethylene

**Figure 6:**
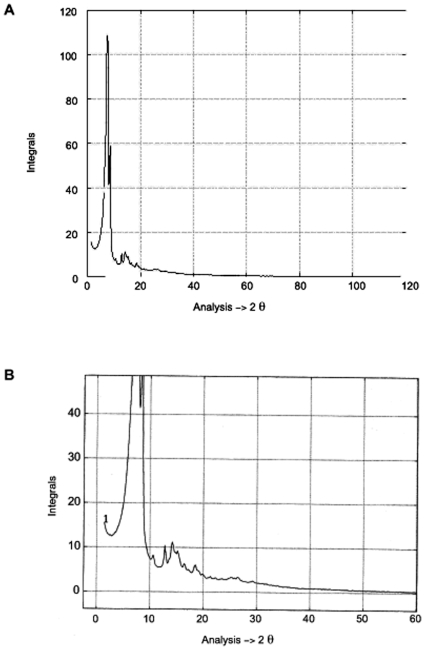
The complete Fourier space image from bulk polyethylene. A-This diagram was obtained by an anomalous diffractometer with an Ag target. λ_Ag Kα_ = 0.5609 Å, K∼20 Å^−1^, 2θ range 0.2–120°. Some of the selective reflections were indexed by Bunn and others, without taking the coherent diffuse scattering into consideration. They supposed an orthorhombic cell as a unit of periodic atomic network of polyethylene. Since discrepancy between calculated and measure intensities, indicated the inconsistency of the coherent model. B-This figure is the zoomed section of 2θ, between 0 to 60°. It can be clearly seen that the coherent diffuse scattering cannot be neglected.

**Figure 7:**
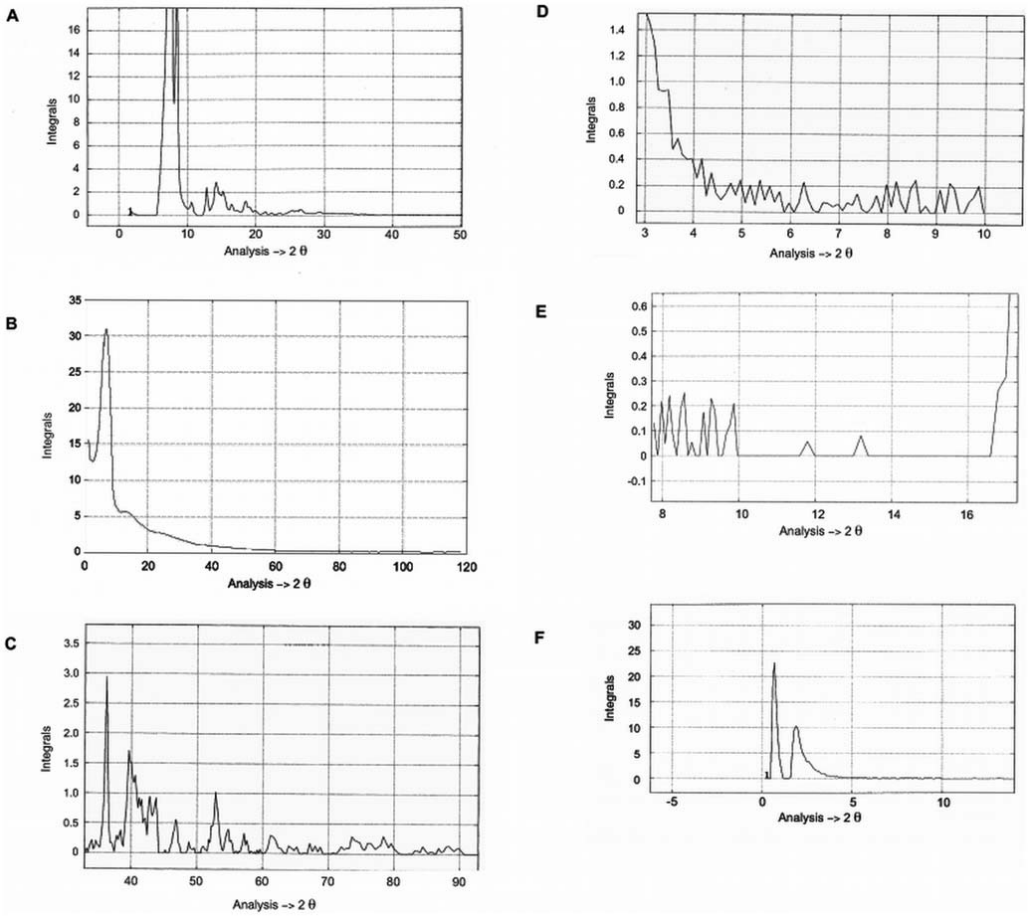
The complete image analysis of polyethylene Fourier space. A- I_s_ (2θ) is the line spectrum at λ = 0.5609 Å. B- I_c_ (2θ)- The x-ray pattern of bulk polyethylene after separation of selective reflections from the coherent diffuse scattering showing the spectrum of coherent diffuse scattering at λ = 0.5609 Å. C-.The diagram was obtained at λ = 1.54 Å and the zoomed portion shows the numerous selective lines. D to F-The diagrams were obtained at λ = 1.54 Å (Cu K_α_) and the zoomed portion shows the numerous selective lines before the first Bragg lines d_(110)_ = 4.106 Å. 7F indicates two distances: 2θ = 0.670°, d = 130 Å and 2θ = 1.870°, d = 46.95 Å respectively. These lines can be only observed by our separation method that is based on the experimental elimination of the undesirable and incoherent radiations.

a – analysis of complete image: Figures 6A and B show a (θ–2θ) scan of bulk polyethylene at room temperature obtained by anomalous diffractometry. This image indicated that the selective reflections were superimposed on the coherent diffused scattering (Figure 6B) (zoom of Figure 6A). Our separation procedure separated the selective reflections from the coherent diffused scattering. Therefore, we were able to analyse each spectrum separately (Figures 7A and 7B)

#### Spectrum of Selective Reflections: Line Spectra

Figures 7A and C show that by excluding the coherent diffuse scattering we obtained line spectra similar to any x-ray pattern of polycrystalline materials (Figure 1). We calculated the “d” spacings by the Bragg equation. The “d” values were compared to the d_(hkl)_ spacings calculated from orthorhombic structure with the lattice parameter, a_0_ = 7.40, b_0_ = 4.93, c_0_ = 2.534 Å proposed by Bunn [22].

Table 1 shows the d_hkl_ as interplanar spacings of (hkl) family planes in the crystalline lattice model. Our calculated d values obtained from nλ = 2dsin θ were similar to d_hkl_ as an optical ruled grating [27], in the bulk polyethylene were the same. Except those of the missing reflections, which were hidden behind the background of the traditional x-ray image (see Figures 3A and B Guinier Pattern). Moreover, Table 1 illustrates the relative integrated intensities obtained by anomalous diffractometry did not match those obtained by photographic measurements [22]. However, before the first Bragg line (d_(110)_ = 4.106 Å), reported by Bunn, numerous selective reflections were observed on the line spectra (Figure 7D and 7E). These extra lines were also observed in other substances with intermediary structures such as Al–Mn alloy, which is an archetypal quasi-crystal material [16].

The discrepancies between the calculated and the measured intensities reported by Bunn resulted from the crystalline model, the ‘oriented samples’ and the absence of the first powder lines. These resulted in a doubtful structural determination by the powder method. It is clear that even if we ignored the coherent diffuse scattering performed in case of polymer characterisation, the principle of indexing x-ray patterns needed to be respected. That was the vital factor that determined the indexibility of powder pattern. These essential factors determined the accuracy of the position (θ), the profile and the integrated intensity of diffracted lines reflected from the polycrystalline grains. We concluded that the full Fourier space image of polyethylene could not be consistent with Bunn's crystalline model, which was proposed in terms of simple resolution. This inconsistency with x-ray diffraction diagram can be proven also by the deficiency of density.

b-Deficiency of density in crystal model of bulk polyethylene (testing the geometrical model):

The measured density, d, of bulk polyethylene by pyknometry method was always below the theoretical density, ρ_0_ (crystal density), that was calculated from the crystal unit cell volume, with two ethylenes in the unit cell of Bunn's model.

The difference between d = 0.92 g/cm^3^ and ρ_0cal_ = 1.0259 g/cm^3^ from the crystal structure it was interpreted that polyethylene was rarely, if ever fully crystalline. In other words, the density defect predicted the polymer's atomic structure as a two-phase model. This model was considered as a simplified picture of a polymer structure where a crystalline domain is interspersed with the amorphous region [5]. In this particular case amorphous was defined as non-crysalline and was supposed to be less compact material than crystals. This phenomenon was suggested to be the cause of the density deficiency.

On this ground, the degree of crystallinity (x_c_) has been defined for indicating the numerical measurements of order present in the polyethylene sample. Differences in experimental techniques could, thus give rise to different estimates of crystallinity, so that elusive variables were considered to be the important effect on the polymer's physical property [12], [21].

To confirm the above argument we showed experimentally, that classical resolving procedures (excluding the background and considering only the lines as Bragg reflections), of x-ray patterns of samples have resulted in misleading information. For instance, the whole image of reciprocal space of non-ideal solid solution, Al–Cu alloy, was obtained by anomalous diffractometry, the image consisted of selective reflections superimposed on a coherent diffuse scattering [17]. If we had used the traditional procedure of resolving the powder lines would only be indexed as pure Al with lattice parameter a_0_ = 4.050 Å. However, this alloy was recognised as an age hardening alloy since the discovery of the Guinier-Preston zone. Its mechanical behaviour was totally different from pure aluminium, which easily submits to plastic deformation. The result of this original structural investigation showed that the two-phase model was inconsistent with the total Fourier space image of the Al–Cu alloy when one part of the intensity (coherent diffuse scattering) was excluded. Thus the knowledge of average perfectly periodic structure as an imaginary periodic model was not sufficient to explain the physical or mechanical behaviour of matter as we showed in the results obtained from Al–Cu.

Based on this example it can be clearly seen that polyethylene's atomic structure was too complex to be solved by a simple approach of resolving the powder pattern and indexing only the powder lines, as the crystalline part.

We therefore turned to more powerful methods of interpretation of whole reciprocal space, such as our separation procedures.

To establish a more realistic structural characterization of polyethylene its coherent diffuse scattering spectra were analysed.

### The analysis and interpretation of the Coherent Diffused Scattering Spectra

The reduced interference function, F (K) - Figure 7B shows the intensity variation of the coherent scattering versus the diffraction angle I_c_ (2θ) separated from the selective reflections. To interpret the coherent continuous diffused scattering at first approximation it can be imagined that the atoms were distributed as a monatomic perfect gas. In this case, the calculated diffused scattering curve decreased as a (2θ) increased. This curve would be similar to the form factors of perfect monatomic gases. We considered this curve as a reference to the disordered state, as we mentioned above, however, the modulated features of diffused scattering in the Guinier pattern (Figures 3A and B) indicated that these random atoms were not disordered as a perfect gas. They were tightly packed together to form a local order, known as the short-range order. This signified that the coherent diffuse scattering spectra primarily probed the atomic correlation in the first and second coordination shells. The curve would be considered as the form factor of the atoms deviated from the regular site.

**Figure 8:**
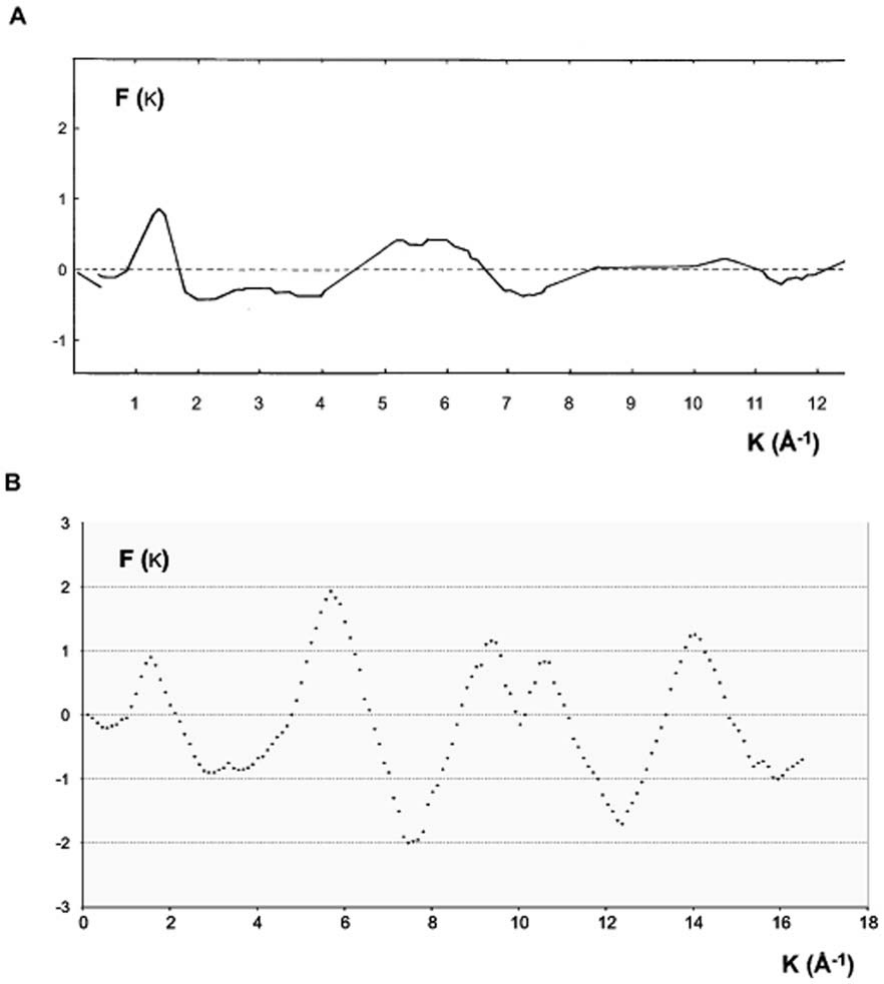
The reduced interference function, F(k), derived from the coherent diffuse scattering, I_c_ (2θ). A. The analysis of F(k) shows a quick dampening which is different to the dampening of the liquid state. Also F(k) tended towards zero due to the absence of correlation between the position of any molecule. B. Contrary to the interference function of bulk polyethylene, the liquid state polyethylene F (k) curve showed intense oscillations beyond k = 7 Å^−1^ [36].

Figure 8A shows the reduced interference function, F (k), derived from the coherent diffuse scattering, I_c_ (2θ) (Figure 7B). The principal peak at k_1_ = 1.39 Å^−1^, was associated with the far atomic correlation shell. The half maximum breath of this peak Δk_1_ = 0.56 Å^−1^ (with λ = 1.54 Å, 2θ = 7.87°) indicated the degree of randomness of the furthest atomic shell. When Δk_1_ was compared to the principal peak Δk_1_ = 0.37 Å^−1^ of interference function of molten polyethylene at 1.40 Å [36] it indicated that the interaction between the furthest atomic shell of solid polyethylene was smaller than its liquid state at T = 413 K (Figure 8B) [36]. Figure 8A illustrates two other peaks, the first one at 2.9 Å^−1^, and the other with a higher intensity at k_3_ = 5.3 Å^−1^ (Δk_3_ = 1.62 Å^−1^). Beyond these two peaks the oscillations of F(k) obtained from bulk polyethylene dampened out very quickly. No further oscillations were recorded. F(k) tended towards zero due to the absence of correlation between the position of any molecule. Contrary to the interference function of bulk polyethylene, the liquid state polyethylene, F(k), curve [36] showed intense oscillations beyond k = 7 Å^−1^ (Figure 8B). These intense oscillations suggested the presence of correlation in the medium range structure of molten polyethylene. However, for a clearer understanding of the short-range order we introduced the mathematical concept of radial distribution function.

### Interpretation of Reduced Radial Distribution Function W(r), comparison of the result with chain model (Bunn's and Flory's model)

**Figure 9:**
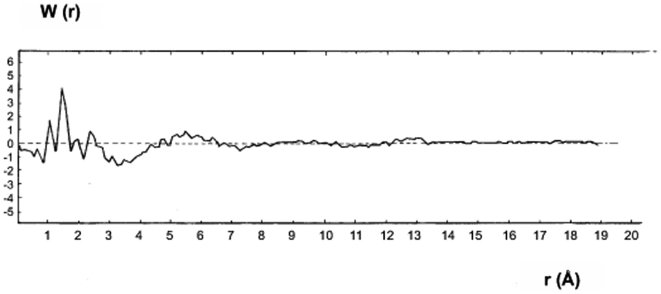
The reduced radial distribution function derived by Fourier transformation of F(k). Each maxima gives the probability of average distance of two different sites of c–c and the absence of the maxima at r = √2×1.48 = 2.093 Å. This singularity indicates the absence of crystalline clusters. The curve below the first peak of W(r) indicates a slope of “−1” that is used to verify the macroscopic density.

Figure 9 shows the radial distribution function, which provided information on the environment of each atom from the atomic network. It is well known that the Debye equation (1) applied only to monatomic materials. This was nearly the case of polyethylene, since x-ray scattering by hydrogen was negligible, for the analysis of W(r) only the scattering factor of carbon were taken into consideration as was mentioned before. Consequently, the interatomic carbon-carbon distances of polyethylene were obtained directly from this function. As shown previously, this function, W(r), was derived from the Fourier transform of the reduced interference function, F (k).

The details of the short-range order were revealed by this curve. The curve was determined at a large K with interval 0.01<K<20 Å^−1^ .The maximum at 1.48 Å in W(r) may be attributed to the atomic diameter of carbon-carbon, c_1_−c_2_ = r_1_ = 1.48 Å. This principal peak r_1_ was sharp and well resolved. The sharpness of this peak (FWHM = Δr_1_ = 0.22 Å) indicated that the distances between the nearest carbon atoms were well defined and the area under the peak was directly attributed to the nearest atoms. The maximum corresponded to r_2_ = 2.41 Å and the atomic diameter of r_2_ = c_1_−c_3_. The ratio of these two maxima: c_1_−c_2_ = 1.48 Å and c_1_−c_3_ = 2.41 Å, r_2_/r_1_ = 1.628, approximately corresponded to √8/√3 = 1.633.

This value was expected for four carbon atoms with a tetrahedral arrangement. The analysis of this result showed for the first time that the regular tetrahedron in diamond was preserved in the carbon atomic network of bulk polyethylene.

This value was expected for three atoms bonded in a tetrahedral configuration, where they formed an isosceles triangle with a tetrahedral angle θ = 109°.

By analysing the coherent diffuse scattering spectra, the geometrical assignment of atoms that surrounded all other atoms was obtained. This geometrical assignment was assessed regardless of whether the polyethylene was a crystalline or amorphous state.

### Comparison of models with experimental data

#### The linear zigzag chain model

To interpret the other maxima of W(r) curve, we assumed that the disordered carbon atoms assigned as the model of polymeric molecular chains of the diamond structure, where the elementary units in the one -dimensional network contained one atom and one bond (Figure 5) [21].

Therefore polyethylene had the same tetrahedral valencies as were associated with carbon atoms in the diamond network with an sp^3^ bond configuration. In such a case, we could assume that the backbone of carbon atoms formed a linear zigzag chain, along with its, one-dimensional topology, similar to the diamond molecular chains. In this case the valence bond was l.48 Å, instead of 1.5533 Å interatomic distances of c–c and valence angle of 109° instead 109°28'. Since three atoms of the elementary unit form a triangle we can write sin θ = r_2_/2r_1_ = 0.814, where θ/2 = 54.489°. By trigonometry the distance of c_1_−c_4_ = 3.72 Å (r_3_) was calculated, which was the position of the third neighbouring atom. From the chain model the position of the fourth neighbouring atom in the planar carbon chain was assessed to be twice the period of the zigzag c_1_−c_5_ = 2×2.41 = 4.82 Å (r_4_). The correlation between these calculated carbon-carbon distances in planar chain and the other maxima in W(r) curve showed that the deviated atoms in solid polyethylene were far from being concatenated as a linear zigzag chain.

On the other hand, by carefully studying the behaviour of reduced radial distribution function, W(r), (Figure 9), a very broad asymmetrical peak between 3.60 to 4.30 Å was noticed. Therefore, the area under such large peak did not render the number of neighbouring atoms, compared to the principal peak, n_c_ = 1.92∼2. Furthermore, the elevated value related to Δr indicated large fluctuations for bond angle and bond valence lengths. By considering the same chain as the linear chain position the 5^th^ and 7^th^ atoms were determined from the reference atoms. As mentioned above r_6_ was calculated to be, three fold the zigzag period (r_6_ = c_1_−c_7_ = 3×2.41 = 7.23 Å). The two maxima in Figure 9, W(r) at 4.78 Å and 7.10 Å, may be approximately attributed to the calculated distances of r_4_ and r_6_ respectively. However the area under these small peaks did not confirm the principle characteristic of a planar zigzag chain model with a co-ordination number of n_c_ = 2. Moreover, the calculated distances for atom 8 (r_7_), c_1_−c_7_ = 8.71 Å was beyond 8 Å. In the W(r) such a structure was not present. Figure 9 shows that the configurated structure after the 6th atom was also absent.

**Figure 10:**
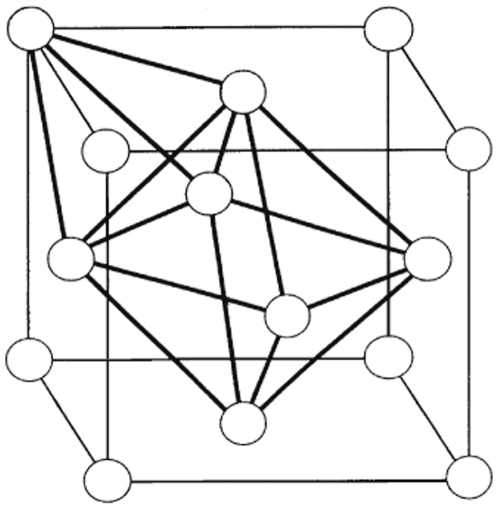
The construction of a crystal structure (f.c.c) by combining the tetrahedral building block and the octahedral configuration. The f.c.c crystal structure can be constructed by combining the tetrahedral building block and octahedral configuration to fill the space. The absence of octahedron geometry prevents crystal nucleation.

Moreover, it is known that a regular tetrahedral arrangement of atoms can be incorporated into periodic space, filling arrangement, only when combined with other types of configuration (like an octahedron) to obtain f.c.c. structures (Figure 10.) The absence of the singularity at √2r atomic diameter indicated that a crystalline cluster was not present. The study of the well resolved region of W(r), did not show a maximum correlation to a distance at √2×1.48 = 2.093A°.

On the other hand, we observed on the left hand side of the principle peak (1.48 Å), an interatomic distance of 1.10 Å, which was surprisingly much smaller than the covalent radius of a single carbon bond. To verify, if this small distance was an artefact, we changed the upper limit of the integration (k_max_) of the reduced interference function (see Equation 3), F(k) in Figure 8A.

This operation did not produce a quantitative change at the maximum of the W(r) curve. We also observed peaks on the right hand side of principle peak in W(r). The first one corresponded to the distances, r'_2_ = 1.96 Å. The ratio of these two interatomic distances (r'_1_ = 1.10 Å and r'_2_ = 1.96 Å) was about √3. This corresponded to the distance across two edge-sharing equilateral triangles.

Therefore it could be considered that the atoms of carbon formed a tetrahedral arrangement with the length of valence bond l = 1.10 Å and base 1.96 Å. If we always considered the chain as a linear zigzag chain, r'_2_ = 1.96 Å could be considered as the base, with a bond angle θ' = 125°. In the network of deviated atoms of polyethylene, this configuration was chosen as an elementary unit of other hypothetical planar zigzag chains.

If this assumption were true, the position of the fourth atom could be identified by trigonometry calculation. A sharp peak in W(r) that corresponded to the calculated r_3_' distance c_1_'−c_4_' = 2.92 Å. The absence of a peak corresponded to the 3^rd^ nearest neighbouring distance in the W(r). The presence of a very large peak in form of the flat region of 3.60–4.30 Å, that corresponded to the 4^th^ nearest neighbouring distance (r_4_' = c_1_'−c_5_' = 2×1.96 = 3.92 Å), was the additional evidence against the planar zigzag model.

But there are others peaks at 4.78 Å, 5.89 Å, 6.97 Å and 7.89 Å in the W(r) curve, which corresponded to the calculated distances of bond length for atoms 6, 7, 8 and 9 considering the planar zigzag chain were r'_5_ = c'_1_−c'_6_ = 4.93 Å, r'_6_ = c'_1_−c'_7_ = 5.88 Å, r'_7_ = c'_1_−c'_8_ = 6.88 Å and r'_8_ = c'_1_−c'_9_ = 7.8 Å respectively.

The comparison of these calculated bond length with the peak positions suggested that these peaks could be attributed to the 5^th^, 6^th^, 7^th^, and 8^th^ nearest neighbouring peaks. On the other hand, we know that such one-dimensional network model was characterised by its co-ordination number, n_c_ = 2, since the areas under these small peaks did not correlate. The maxima in W(r) cannot therefore, be explained by such a hypothetical linear zigzag chain model. In addition to the flat region between 3.60 to 4.30 Å, we observed another broad peak with half maximum Δr = 0.50 Å which showed a large fluctuation of the bond length that varied between 2.10 to 3.05 Å. The maximum envelope of these different distances was at 2.71 Å in the W(r) curve. In order to explain the flat region between 3.60 Å to 4.30 Å and this large peak, the rotation of the bonds was considered. Flory proposed a freely rotating chain model was for glassy or disordered polymers [37], [38], [39].

#### A random coil model

**Figure 11:**
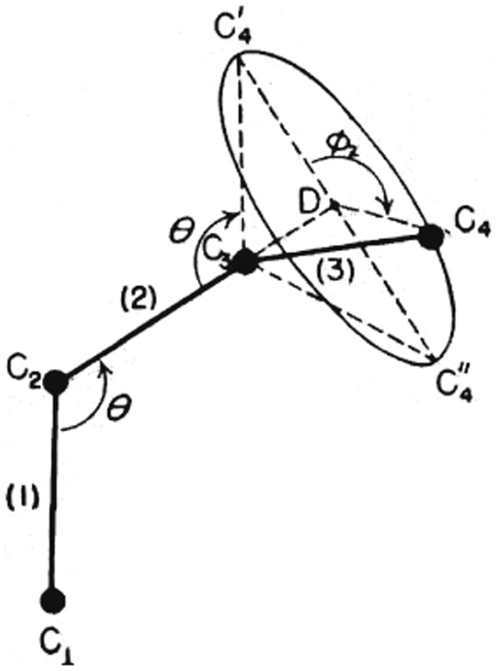
The relation between bond angle “θ” and the rotation angle “ϕ” considering the free rotating chain model for carbon atoms in bulk polyethylene. This scheme was modified from Chapter 10 pg 400 Figure 74 “Principles of Polymer Chemistry” [38].

A random coil model was a model in which the chain adopted a conformation, referred to as the geometrical arrangement of carbon atoms with respect to rotation of chain about a single bond. The essential and striking features of this model were Flory's suggestions that the chain conformation (geometrical arrangement) in the melt were a good approximation unperturbed by presence of neighbouring chains and that the conformation of chain in the melt or glassy polymer were similar to those in diluted solutions. This model was verified by the interatomic distances obtained from the radial distribution function W(r). In this model, the elementary triangle constituted of 3 atoms (1,2,3), the fourth atom that followed atom 3, should be in such a position that it formed the second triangle (2, 3, 4) with reference to the plane (1, 2, 3). Therefore, atom 4 may occur any place on the circle, which was the base of the cone described by the rotation of bond 3 (bond c_3_–c_4_) specified by the rotation angle (ϕ_2_). Here we have assumed that the length (l) of valence bonds in the chain and the valence angle bonds (θ) to be fixed. The position of atom 4, was known on circle c (Figure 11) [39] and *a priori* all the positions of atom 4 were on the circumference of circle c. Similarly bonds 3–4 defined the plane 3, 4, 5 and the direction of bond 4 (not observed in Figure 11). The position of atom 5 with rotation angle of (ϕ_3_) around bond 3 relative to bonds 2 and 3 was also defined.

The chain conformation of a fixed θ, l and an arbitrary small variance of ϕ, (Δϕ) could be determined in this manner.

In other words, this arbitrary Δϕ determined the chain conformation, for such a model where θ was assumed to be fixed and l a *freely rotating chain*. According to this model, when the bond angle θ = 109° and bond length l_1_ = 1.48 Å was considered to be fixed the distance c_1_−c_4_ = (6.48+7.26 cos^2^ϕ/2)^1/2^ was determined relative to the rotation angle. If we assumed that atom 4, scanned the circle by steps of ϕ = ϕ_2_+Δϕ_i_ we obtained the different lengths (l) for valence bonds with variable directions. These vectors express the probable positions of atom 4 on circle c. When the angle ϕ = ϕ_2_+Δϕ_i_ varies from ϕ = 0 to ϕ = 2π it was obvious that for ϕ = 0, atom 4 was situated on the same plane defined by bonds 1 and 2. In this case, we obtained the same value for the distance c_1_–c_4_ calculated from planar chain model c_1_−c_4_ = 3.71 Å. Once again, this value did not match with any maximum of the radial distribution function derived from the deviated atoms. By changing ϕ each 5 degrees, Δϕ = 5, we obtained the following values for the length (l) of the valence bonds (Table 2).

**Table 2:** The different length for valence bond with variable direction for l_1–2_ = 1.48A° and θ = 109°.

**ϕ**	**5**	**10**	**15**	**20**	**25**	**30**	**35**	**40**	**45**	**50**
l = c_1_–c_4_ Å	3.71	3.70	3.69	3.68	3.66	3.64	3.61	3.59	3.56	3.52
**ϕ**	**55**	**60**	**65**	**70**	**75**	**80**	**85**	**90**	**95**	**100**
l = c_1_–c_4_ Å	3.49	3.45	3.41	3.37	3.32	3.28	3.23	3.18	3.13	3.08

As shown in Table 2, the continuous vectorial variation of bond 3 is explained by flatness of region 3.60 to 3.40 Å in W (r) curve. Moreover, these different distances of c_1_–c_4_ were covered by a large envelop, in which they were related to continuum variation of ϕ = ϕ_2_+Δϕ_i_.

Therefore, atom 4 occurred at N positions at circle (c_4_) and atom 5 on circle c_5_ specified by rotation angle ϕ_3_, ϕ_4_ (ϕ_4_ is the angle between plans 2, 3, 4, and 3,4,5). These are N^2^ conformations that result in the probable position for the 4^th^ neighbouring atoms. The total numbers of possible conformation may attribute to bond 4(c_4_−c_5_). The assumption of such a model, the position of the nth atom in the network of polyethylene is fixed by n−3 rotations or n−3 degrees of liberty. The determination of the position of neighbouring atoms became more complex. Such overwhelming complexities of atomic arrangement of deviated atoms was confirmed by the feature of radial distribution function W(r), derived from coherent diffuse scattering I_c_ (k). The other structural information obtained from W(r), was the damping out of the curve beyond 8 Å. This experimental observation expresses no correlation beyond the first and second atomic shell in core of polyethylene.

To interpret the peak at 2.71 Å in W(r) curve, according to the “freely rotating chain model”, we calculated in the same manner as above the probable position of atom 4 on the circle (c'_4_).

If we considered the fixed bond length l' = 1.10 Å and bond angle θ' = 125°27', by changing ϕ' = ϕ'_2_+Δ ϕ_i_ we obtained the values in Table 3. The distance 2.98 Å corresponded to the ϕ' = 0 or 2π degree. Obviously not all maxima correlated to the distance. Therefore, this result showed carbon atoms in bulk polyethylene, were not arranged in the planar zigzag chains. The position of atom 4 was determined by the distance c'_1_−c'_4_ = 2.71 Å with a rotation angle of ϕ'_2_ = 90 (Table 3).

**Table 3:** The different length for valence bond with variable direction for l'_1–2_ = 1.10 Å and θ' = 125°.27'.

ϕ	0	2π	30	40	60	80	90	100
l'_1–4_ = c'_1_−c'_4_	2.98	2.98	2.94	2.92	2.84	2.75	2.70	2.65

This distance corresponded to the maximum 2.71 Å in W(r) curve. The broadness of this peak, Δr = 0.50 Å indicated the distribution of different distances of probable positions on the circle (c'_4_). Indeed, the peak at 2.71 Å in W(r) was the maximum of the envelope of the different values of bond length of atom 4, in the chain of carbon with valence angle of θ = 125°27'. Hence, there are N probable positions for atom 4, between 2.15 Å up to 3.05 Å and, N^2^, positions for atom 5 in the case of the chains with bond length of l'_1–2_ = 1.10 Å.

By the analysis of coherent diffuse scattering we deduced that there were two types of carbon atoms, which formed two random nets, which the atomic network of polyethylene was formed. The geometrical factors of each chain were different; one chain net had the covalent bond of c_1_−c_2_ = 1.48 Å with valance angle of θ = 109° and another chain with covalent bond c'_1_−c'_2_ = 1.10 Å with θ' = 125°27'. Therefore according to the experimental results the deviated atoms were distributed as a heap of covalent molecules of carbon atoms.

### Primacy of short-range order: geometrical factor and chemical ordering

The experimental results obtained from anomalous diffractometry determined the predominance of geometry of local order determination in the atomic network of bulk polyethylene compared to lattice structure determination. We have shown that the deviated atoms, in the core of bulk polyethylene, were not distributed in a “disordered” manner but

each carbon atom with bond length, l = 1.48 Å, was surrounded by four atoms at corners of regular tetrahedron, θ = 109° and four atoms with a bond length shorter, l' = 1.10 Å, formed a non-regular tetrahedron, θ' = 125°27'. The notable common structure feature with diamond was the geometry of local topology that meant the geometry arrangement of four carbon atoms in the atomic network, was a tetrahedron around an atom. Therefore, the short-range order (first co-ordination shell) of bulk polyethylene was reminiscence of diamond. But the analysis of line spectrum did not show the long-range order of diamond. Since the analysis of radial distribution function, W(r), indicated an atomic network of bulk polyethylene consisting of the small heaps of tetrahedral configuration of carbon atoms with two different bond lengths differing from atomic radius of crystalline carbon diamond. We mentioned that this type of atomic arrangement could not grow as a crystalline phase. This result might explain why the polyethylene liquid could be super-cooled, resisted to crystal nucleation when it was solidified and it had non-abrupt melting points without a first order phase transition [40].

These results illustrated the reason for determining the short-range order, which in turn indicated the geometry of local order and chemical ordering. These parameters were of paramount importance in polymer structural studies.

In other words, a thorough determination of the short-range order was instrumental in reducing expenditure of theoretical effort by at least a limiting number of meaningful structural models to which the theoretical calculation may be applied.

In this respect, we compared c–c distances of the isolated planar chain with the results obtained from radial distribution function W(r).

The full image of reciprocal space obtained by anomalous diffractometry illustrated the inconsistency of the traditional models (the two phase concept and the crystalline defect concept). These two models led to defining a macroscopic concept for polymer structure known as morphology. However, from the morphology the geometrical factor and the bond shortening cannot be identified. It was important to have an understanding of the physical properties of polymers, in order to explain the bond length shortening of l' = 1.10 Å and other relative carbon-carbon distances.

From the atomic structural interpretation and the electronic hybridisation structure, it can be assumed the carbon crystallised in the diamond structure. In such extreme cases the Goldschmidt atomic and covalent radii of c–c were the same (c–c = 1.5433/2 Å). The idea that interatomic distances or bond lengths interpreted in terms of covalent radii was initially introduced for carbon. In this case the single bond radius was derived from the crystalline structure of diamond. This bond radius agreed with c–c distances in other compounds that were supposedly crystalline substances. An example of this in literature was bulk polyethylene [41]. However, our data did not coincide with the c–c radius mentioned. The double and triple covalent bond lengths were determined experimentally. Typically, the bond-lengths in single, double and triple bonds decreased with increasing bond numbers (n). Pauling combined the effect of bond order and resonance in a single equation:

R (1)−R (n) = 0.300 log n, where the relation between atomic radius and bond type was explained. n is the number of share electron pairs, (R1) is the covalent radius of a normal single covalent bond and R (n) is the radius in a structure with ν normal single covalent bonds that resonate among N position with then n = ν/N. That is each atom contributes one electron and they were shared equally between the two atoms. This equation was really based on the interatomic radius in carbon compounds and its validity when applied to metallic substances was disputed [41]. We have shown experimentally that the validity of the calculated Pauling single bond radius for metallic alloys [17] and evidenced that the shorter bond-length l' = 1.10 Å may be equivalent to the radius of double bonded carbon atoms in the network of bulk polyethylene.

Nevertheless, despite the difference of opinion on the general application of the Pauling equation there was a uniform agreement that the cohesive forces in alloys were closely related to those of covalent bonds [41].

We have previously reported the shortening of interatomic distances in non-ideal solid solution (Al–Cu) [17]. Three interatomic distances were deduced from the maxima 2.87 Å, 2.71 and 2.17 Å of the radial distribution function W(r), derived from the coherent diffuse scattering of this alloy. The first distance corresponded to the interatomic distance of Al–Al (2.86 Å); the second to the Cu–Al and the third to the calculated Pauling single bond radius (r_Cu_ = 1.17/2 Å) The latter distance was shorter than the Cu–Cu interatomic distance (Cu–Cu = 2.56 Å). The other maxima of the atomic distribution function W(r) curve indicated the Pauling single bond radius for Cu and Goldschmidt radius for Al (r_Al_ = 2.86/2 Å); they each formed a tetrahedron which linked together to form two types of non-crystalline icosahedral cluster which were joined together to form a pentagonal chains with chemical ordering. These chains prevented the formation of crystalline materials and induced an intermediary state of order [16], [17], [18]. The interpretation of whole Fourier space image of these pentagonal chains showed that indexing the diffraction line without considering the coherent background is an inaccurate characterisation of materials.

The precise examination of line spectrum indicated that the reflection lines could be indexed as the Bragg reflection of pure crystalline aluminium, up to very large K = 20 Å^−1^, with fcc structure and lattice parameters a_0_ = 4.050 Å. As mentioned above the mechanical behaviour of pure Al was totally different from this alloy. Pure Al submitted easily to plastic deformation however this alloy did not. Therefore the sample was recognized as a hardening alloy [42]. The example of Al–Cu alloy showed that reductionism in structural determination resulted in characterising a hard alloy as soft pure aluminium. This original investigation pointed out the origin and the condition of the extra stabilisation was due to the resonance energy of a strong bond with smaller interatomic distances (metallic alloys 1.17 Å, and polyethylene 1.10 Å) than a weaker bond.

The coherent background had a considerable influence on the mechanical behaviour or physical properties that were determined by the refined approach of anomalous diffractometry method. Thus a sample with a complex structure would be characterised more precisely by local geometry of irregularity (nanometer scale). By analogy indexing the line spectrum of polyethylene and excluding the coherent diffuse scattering yielded to the artificial concept of orthorhombic crystal structure reported by Bunn [22].

Inherently the degree of crystallinity (x_c_) issued from the two-phase concept or the highly defective crystal model was not used to determine and understand the mechanical behaviour of polyethylene.

The fact that the deviated carbon atoms in atomic network were bonded with a tetrahedron environment prevented the formation of crystalline structure. Since the oscillations of interference function of disordered atoms F (k) dampened out quickly beyond 8 Å^−1^. This curve also indicated that for medium range order there was a poor correlation. Moreover, comparing the F (k) derived from coherent diffuse scattering (Figure 8A) with reduced interference function of molten polyethylene (at 413K, Figure 8B), pointed out the superiority of the degree of randomness of deviated atoms in bulk polyethylene versus the liquid state. This state of randomness was considered as a reference of disorder in materials with an intermediate order. Previously, the same phenomenon was shown in electronic polymers (polyaniline) [25].

Furthermore, the preservation of the local tetrahedron geometry of deviated atoms was strong evidence that the paracrystalline model, a pattern using sloppy lattice instead of a perfect lattice network of polyethylene, did not fit with the atomic network of polyethylene [43].

It was therefore more realistic to consider polyethylene as a substance with intermediate structure situated between the extreme types of perfect order or disorder [9].

In conclusion, from the precise structural details in this study essential generalities such as geometrical factor and macroscopic density were determined. From these generalities a “true” picture of the atomic arrangements of bulk polyethylene were obtained. However there is still a need to study how the irregular local geometry of polyethylene may be packed to occupy the space that has a high packing density of 0.630. The packing density of random close packing of hard spheres was 0.637 [44].

In the case of polyethylene with an intermediate structure we were still unable to characterise its irregular array in formal mathematical terms.

Bernal and Finny took the first step to express the need for the science of “statistical geometry” for studying the liquid state [44], [45]. We adapted this school of thought, which was characterised in terms of simple geometrical elements with five-fold symmetry and proposed a new picture for the atomic arrangements of amorphous alloy [43], [44] and metallic alloys with an intermediary structure [16], [17], [18].

Substances with an intermediary structure are not rare exceptions in nature. This powerful method of investigation permitted us to study the complex structure formed by large and flexible chained molecules, which are also present in the biological sciences or various other polymers.
